# Development and Evaluation of a UAV-Photogrammetry System for Precise 3D Environmental Modeling

**DOI:** 10.3390/s151127493

**Published:** 2015-10-30

**Authors:** Mozhdeh Shahbazi, Gunho Sohn, Jérôme Théau, Patrick Menard

**Affiliations:** 1 Department of Applied Geomatics, Université de Sherbrooke, 2500 Boulevard de l’Université, Building A6, Sherbrooke, QC J1K 2R1, Canada; E-Mail: jerome.theau@usherbrooke.ca; 2 Department of Earth and Space Science and Engineering, York University, 4700 Keele Street, Petrie Science & Engineering Building, Toronto, ON M3J 1P3, Canada; E-Mail: gsohn@yorku.ca; 3 Centre de géomatique du Québec, 534 Jacques-Cartier Est, Building G, Chicoutimi, QC G7H 1Z6, Canada; E-Mail: pmenard@cgq.qc.ca

**Keywords:** UAV, modeling, photogrammetry, calibration, georeferencing, ground control point, mine

## Abstract

The specific requirements of UAV-photogrammetry necessitate particular solutions for system development, which have mostly been ignored or not assessed adequately in recent studies. Accordingly, this paper presents the methodological and experimental aspects of correctly implementing a UAV-photogrammetry system. The hardware of the system consists of an electric-powered helicopter, a high-resolution digital camera and an inertial navigation system. The software of the system includes the in-house programs specifically designed for camera calibration, platform calibration, system integration, on-board data acquisition, flight planning and on-the-job self-calibration. The detailed features of the system are discussed, and solutions are proposed in order to enhance the system and its photogrammetric outputs. The developed system is extensively tested for precise modeling of the challenging environment of an open-pit gravel mine. The accuracy of the results is evaluated under various mapping conditions, including direct georeferencing and indirect georeferencing with different numbers, distributions and types of ground control points. Additionally, the effects of imaging configuration and network stability on modeling accuracy are assessed. The experiments demonstrated that 1.55 m horizontal and 3.16 m vertical absolute modeling accuracy could be achieved via direct geo-referencing, which was improved to 0.4 cm and 1.7 cm after indirect geo-referencing.

## 1. Introduction

### 1.1. Background

Unmanned aerial imagery has recently been applied in various domains such as natural resource management, spatial ecology and civil engineering [1,2,3]. Most of these unmanned aerial vehicle (UAV) applications require geospatial information of the environment. Consequently, three-dimensional (3D) environmental modeling via UAV-photogrammetry systems (UAV-PS) has become a matter of growing interest among both researchers and industries. However, a surveying-grade UAV-PS has critical differences from traditional photogrammetry systems, which should be considered carefully in its development and application. The following paragraphs discuss the background of UAV-PSs and the efforts made to evaluate their capacities.

Typically, development of a UAV-PS starts with selecting the platform as well as the imaging and navigation sensors compatible with it. Regarding the platform, the payload capacity, endurance, range, degree of autonomy must be considered. In some studies, pre-packaged UAVs are used, e.g., AscTec Falcon8 [4], Aeryon Scout [5], SenseFly eBee [6]. Such systems offer safety and ease of operation. However, they offer less flexibility regarding sensor selection and adjustment.

Navigation sensors play two roles in a UAV-PS: auto-piloting the platform and determining the exterior orientation (EO) parameters of images. High-grade inertial navigation systems (INS) can be used in order to eliminate the requirement for establishing ground control points (GCPs) and to achieve enough spatial accuracy via direct georeferencing (DG) [7]. However, consumer-grade systems are preferred considering the costs and limitations of access to base stations for differential or real-time-kinematic (RTK) global-positioning-system (GPS) [8,9]. In such systems, different strategies might be taken for increasing the positioning accuracy—e.g., replacing poor-quality GPS elevation data with height measurements from a barometric altimeter [10]. Accuracy of DG depends on the performance of INS components and the accuracy of platform calibration. Moreover, the system-integration scheme is important since it controls the synchronization between imaging and navigation sensors. Depending on flight speed and accuracy of INS measurements, the delay between camera exposures and their geo-tags can cause serious positioning drifts [7,11].

When indirect georeferencing is performed, considerable care should be given to several factors such as the accuracy of multi-view image matching, on-the-job self-calibration and GCP positioning. Discussed briefly in few studies [10,12,13], the method used to locate GCPs on the images and the configuration of the GCPs are also important factors in determining the final accuracy of indirect georeferencing. Accordingly, the optimum configuration of GCPs required to achieve a certain level of accuracy is a significant concern in the field of UAV-PS. In most of UAV applications, only a minimum number of GCPs in a special configuration and with a limited positioning accuracy can be established. In order to ensure that the results, based on these conditions, can satisfy the accuracy requirements of the application, it is important to have an *a priori* knowledge of the final accuracy.

In terms of imaging sensors, a high-resolution digital visible camera is the key element for photogrammetric mapping. Despite the benefits of non-metric digital cameras such as low price, light weight and high resolution, the instability of their lens and sensor mounts is still a concern in unmanned aerial mapping. Therefore, intrinsic camera calibration must be performed to determine the interior orientation (IO) and distortion parameters of the camera. When metric accuracies are required, offline camera calibration is suggested [14]. However, offline calibration parameters change slightly during the flight due to platform vibrations and instability of camera components [15]. A solution to this problem is to calibrate the camera by adding its systematic errors as additional parameters to aerial block bundle adjustment (BBA), which is known as self-calibration. However, inaccuracy of image observations may influence the calibration parameters as they are all adjusted together with completely unknown parameters such as object-space coordinates of tie points and EO parameters [16]. Thus, motion blur and noise, which are inevitably present in unmanned aerial images, affect the accuracy of calibration. Besides, the numerical stability of self-calibration decreases highly depending on the aerial imaging configuration. Therefore, careful solutions are required to address the issues of on-the-job self-calibration for unmanned aerial imagery.

### 1.2. Environmental Application

Regarding the environmental application, the system of this study was applied for gravel-pit surveying and volumetric change measurement. This environment was selected because of two reasons. Firstly, open-pit mines provide a challenging environment for 3D modeling. That is, considerable scale variations are introduced to the images due to the low altitude of platform in comparison with the terrain relief [17]. Secondly, there are several mining and geological applications which require high-resolution accurate 3D information of open-pit mines, e.g., geotechnical risk assessment. Previous studies have shown that the topographic data must provide a ground resolution of 1–3 cm in order to predict hazardous events such as ground subsidence, slope instability and landslides [18]. Furthermore, mining companies have to quantify the amount of extracted mass and stocked material regularly. The map scale required for volumetric measurement in earthworks is usually between 1:4000 and 1:10,000 [19]. Considering the requirements of mining applications, including spatial and temporal resolution, speed of measurement and safety criteria, unmanned aerial systems can be better solutions for mine mapping in comparison with traditional terrestrial surveying techniques. This can be noticed by the significant increase in use of UAV-PSs in mining applications during the last few years [20,21,22].

### 1.3. Objectives and Assumptions

This paper presents the details of development and implementation of a UAV-PS. In addition to general aspects of the development, the main focus of this study is to discuss the issues and to perform the experiments that are usually ignored or not thoroughly addressed for UAV-PSs. First, the paper concentrates on the procedures for camera and platform calibration as well as system integration. Instead of discussing the regular aspects of calibration, the main focus is on the design of the test-field and automatic target detection assuming that these elements impact the efficiency of calibration significantly. Regarding the system integration, it is assumed that the developed software solution is able to integrate the navigation and imaging sensors accurately without needing any additional mechanism.

Afterwards, the photogrammetric processing workflow is presented. Some aspects of image pre-processing are discussed and their impacts on the accuracy of modeling are investigated. Then, assuming that the accuracy of on-the-job self-calibration is affected by the imaging network, a BBA strategy is suggested to control this adverse effect. This assumption and efficiency of the BBA strategy are also verified. Furthermore, several experiments are designed to assess the effect of GCPs configuration on modeling accuracy. The main assumption that these experiments verify is that a minimum number of GCPs can provide an accuracy level equivalent to the one achievable with redundant number of GCPs under two conditions. First, they are distributed over the whole zone and their visibility in images is maximized. Second, the imaging configuration is proper. That is the imaging configuration ensures scale consistency of the network.

The rest of the paper is structured as follows: first, the equipment is presented. Then, the procedure of system development, including camera calibration, platform calibration and system integration, are discussed in Section 3. Afterwards, Section 4 and Section 5 describe the methodology of data acquisition and data processing. The experiments performed to evaluate the system are presented in Section 6, and the results are discussed in Section 7. At the end, the conclusions and final remarks are presented in Section 8.

## 2. Equipment

### 2.1. Platform

**Figure 1:**
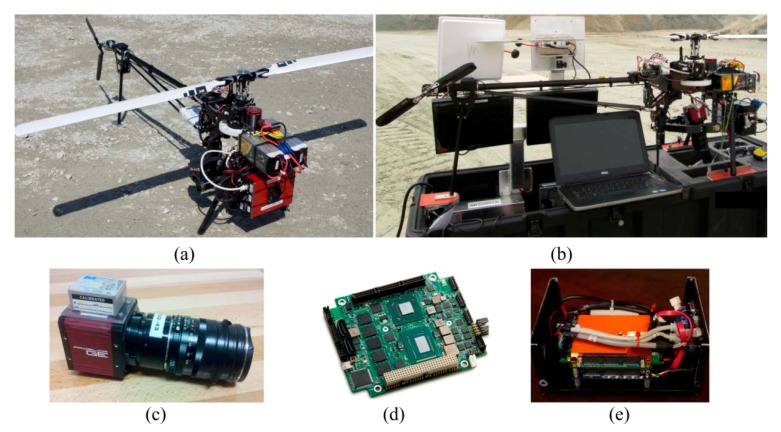
Equipment (**a**) Aerial platform; (**b**) Ground control station; (**c**) INS attached to camera; (**d**) Computer board; (**e**) Computer board and power supply stacked together.

The platform used in this project is a Responder helicopter built by ING Robotic Aviation Inc. (Ottawa, ON, Canada) (Figure 1a). Responder is a vertical take-off & landing UAV which is equipped with a lightweight, carbon-fiber gimbal. This platform has 12 kg payload capacity and cruise operational endurance of 40 min. With our whole independent package of sensors, computer and batteries weighing about 3 kg, the platform could safely fly for 25 min in a day with wind speed of 19 km/h. The platform is equipped with an open-source autopilot—ArduPilot Autopilot Suite. It comes with a portable, compact ground control station to visualize, plan and control autonomous flights (Figure 1b).

### 2.2. Navigation Sensor

The navigation sensor is a GPS-aided INS, MIDGII from Microbotics Inc. (Hampton, VA, USA) (Figure 1c, stacked on the top of the camera). The unit measures pitch and roll with 0.4° and heading (yaw) with 1–2° of accuracy. Its positioning accuracy is 2–5 m depending on availability of wide area augmentation system (WAAS). The output rate of the unit can be extended up to 50 Hz.

### 2.3. Imaging Sensor

The imaging sensor is a GE4900C visible camera (Prosilica, Exton, PA, USA) (Figure 1c). It has a 36.0528 mm × 24.0352 mm sensor at pixel size of 7.4 μm. It is equipped with a 35 mm F-mount lens and supports minimum exposure time of 625 µs. The fact that the camera has a global shutter and charge-coupled-device (CCD) progressive sensor makes the imaging more robust against motion blur, interlacing artifact and read-out delay, which are all essential for UAV-PS [23]. Global shutter controls the incoming light all over the image surface simultaneously. Thus, at any time instance, all photo detectors are either equally closed or equally open. This is in contrast with rolling shutters where exposures move row by row from one side to another side of the image. In the CCD architecture, only one-pixel shift happens to move the charge from image to storage area. Therefore, the readout time and energy consumption decrease considerably. Progressive scanning is also strongly preferred for grabbing moving images, since the images are free of interlacing artifacts caused by the time lag of frame fields.

### 2.4. Onboard Computer

The computer applied in this study is an ultra-small, single-board system (CoreModule 920, ADLINK, San Jose, CA, USA), which is based on a 1.7 GHz Intel Core™ i7 processor (Figure 1d). The board is stacked together with a PC/104 power supply (Figure 1e). The power supply receives 12.8-volt DC input from a 3200 mAh LiFePO4 battery pack. In return, it provides +5 V regulated DC voltage to the computer, +12 V to the camera and +5 V to a fan for cooling the processing unit. With this configuration, the embedded system is capable of acquiring, logging and storing images with a rate of 3 frames per second and navigation data with a rate of 50 Hz during approximately 70 min.

## 3. System Development

### 3.1. Camera Calibration

**Figure 2:**
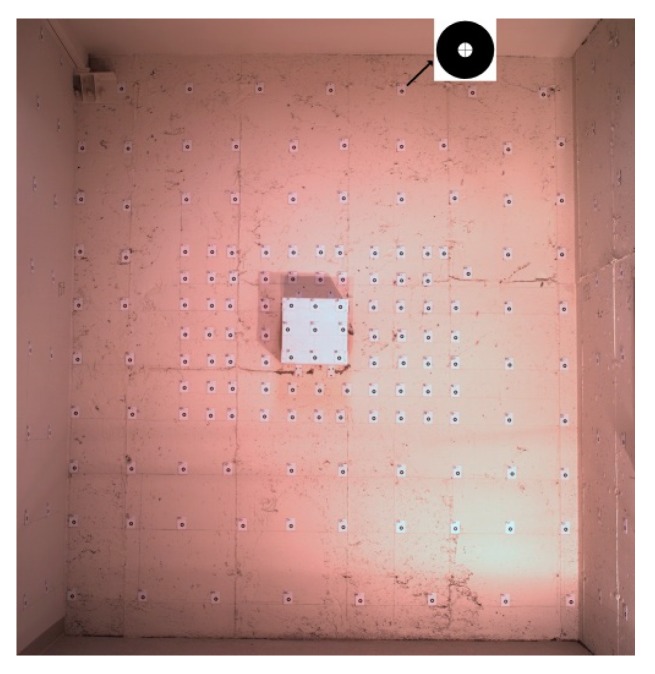
Camera calibration test-field.

In this study, offline camera calibration is performed using a test-field (Figure 2) via a conventional photogrammetric calibration method known as inner-constrained bundle adjustment with additional parameters [24]. In this study, Brown’s additional parameters are applied to model the systematic errors of the camera [25]. Therefore, the camera IO parameters, radial and decentering lens distortions as well as in-plane scale and shear distortions of the sensor [26] are modeled via calibration. As digital camera calibration is a well-studied topic in photogrammetry, detailed theories are avoided here. Instead, other important aspects, including our methodology for test-field design and target detection, are discussed.

#### 3.1.1. Design of the Calibration Test-Field

Two camera parameters determine the size and depth of a test-field: focus distance and field of view. For aerial imaging, camera focus distance is set to infinity so that different altitudes can be covered in the depth of field (DoF) of the camera. However, the focal length extends slightly during focusing (When the focus distance is changed, a small group of elements inside the lens, instead of the whole barrel, is moved to provide the focus. Thus, the focal length changes slightly.). This means that the focus distance should remain fixed all the time. Therefore, it should be ensured that the calibration test-field can provide focused photos at short distances while the focus distance is still set to infinity. Considering Equation (1), the far and near limits of DoF (*H_f_*, *H_n_*) depend on F-number (*d*), focal length (*f*), circle of confusion diameter (*c*) and focus distance (*h*). That is all the objects located between *H_n_* and *H_f_* from the camera can be imaged sharply:

(1) $$H_{f}=h/1-(h-f)c\frac{d}{f^{2}}\text{, }H_{n}=h/1+(h-f)c\frac{d}{f^{2}}$$

If the focus distance is set to infinity (h→∞), then the F-number should be increased largely to provide focus at short ranges. By setting the F-number to its maximum value (*d*_max_), the minimum focus distance can be determined $\left(H_{n_{\min}}\right)$. Thus, the distance of the test-field from the camera, namely the test-field depth, should be larger than $H_{n_{\min}}$. Notice that by maximizing the F-number, the aperture opening (*A*) decreases (Equation (2)), and calibration images become very dark. To compensate this, the exposure time should be increased according to Equation (3). Let *t*_1_ be the minimum exposure time of the camera which is usually selected for aerial imaging to reduce motion blurring artifacts. Accordingly, *A*_1_ is the aperture opening which provides proper illumination for outdoor acquisition in combination with *t*_1_. If *A*_2_ is the aperture opening when maximizing the F-number, then *t*_2_ is the exposure time that should be set to avoid either underexposure or overexposure:

(2) $$A=\pi f^{2}/4d^{2}$$

(3) $$A_{1}t_{1}=A_{2}t_{2}$$

Afterwards, the width and height of the test-field should be determined. It is essential to model the systematic errors based on the distortions observed uniformly across the whole image [24]. Therefore, it should be ensured that the test-field is large enough to cover approximately the whole field of view (FoV) of the camera. The horizontal and vertical angles of FoV ($\alpha_{h},\alpha_{v}$) can be calculated from the sensor size (*W*, *H*) and the focal length (*f*) as in Equation (4). Therefore, the minimum size required for the test-field ($W_{t}\times H_{t}$) can be determined via Equation (5)

(4) $$\alpha_{h}=2\tan^{-1}(W/2f)\text{ , }\alpha_{v}=2\tan^{-1}(H/2f)$$

(5) $$W_{t}=2H_{n_{\min}}\tan(\alpha_{h}/2)\text{ , }H_{t}=2H_{n_{\min}}\tan(\alpha_{v}/2)$$

#### 3.1.2. Target Detection

**Figure 3:**
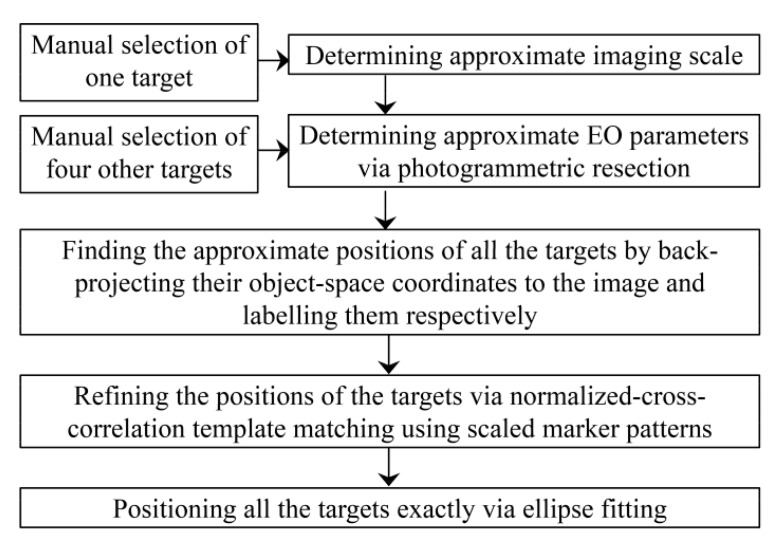
Diagram of the target detection method.

Figure 3 demonstrates the approach of this study for detecting the targets. Unless otherwise indicated, the procedures mentioned in the diagram are fully automatic.

In this study, the targets are designed as black and white rings with crosshairs passing through the circles’ centers. The reason for using circular targets is that once the image of a circle is deformed under any linear transformation, it appears as an ellipse. Then, the techniques of ellipse detection can be applied to position it accurately. The following paragraphs explain the ellipse fitting method developed to determine the accurate positions of the targets.

Assuming that $\left(x_{a},y_{a}\right)$ is the approximate position of a target, a rectangular window is centered at (*x_a_*, *y_a_*). The window is transformed to binary format, and its connected black components are detected. Each closed component represents a candidate region. Let *B* denote the set of all the pixels belonging to the boundary of a candidate region. If the region is actually a target, then its boundary can be modeled as an ellipse with Equation (6):

(6) $${\left(x-x_{o}\right)}^{2}+K{\left(y-y_{o}\right)}^{2}=R^{2}\text{ ; }\forall (x,y)\in B$$

where (*x_o_*, *y_o_*) is the center, R is the flattened radius and K is the aspect ratio of the ellipse. A small percentage of the points belonging to *B* are reserved as checkpoints. Other points are served as observations to determine the ellipse parameters with least squares fitting technique. Once the ellipse is defined mathematically, the checkpoints are validated against the ellipse model. If the fitting error is less than a given threshold, then the candidate region is recognized as a target, and the ellipse center denotes the exact position of the target.

.

### 3.2. Aerial Platform Calibration

The goal of platform calibration is to make sure that EO parameters of images are represented in an earth-centered, earth-fixed (ECEF) reference system, in which the navigation positioning data are represented as well. To this end, the vector between the perspective center of the camera and the center of the INS body-fixed system—known as lever-arm offset—as well as the rotations of the camera coordinate system with respect to the INS system—known as bore-sight angles—should be determined. In this study, the lever-arm offset is ignored. This is due to the fact that the offset between the INS and the camera never exceeds a few centimeters, which is far below the precision of GPS measurements (a few meters).

Attitude outputs from the INS are presented as Euler angles, also known as Cardan. The Cardan consists of three rotations: roll (*ɸ*), pitch (*θ*) and yaw (*ψ*). The rotation matrix $R_{b}^{n}$—composed of Euler angles—rotates vectors from the INS body-fixed coordinates system (*b*) to the local geodetic system (*n*) as in Equation (7). Likewise, the rotation matrix $R_{n}^{e}$—composed of geodetic latitude (*φ*) and longitude (*λ*)—rotates vectors from the local geodetic system to the ECEF system (*e*) as in Equation (8). Therefore, the rotation matrix $R_{b}^{e}$ rotates vectors from the INS body-fixed coordinate system to the ECEF system as in Equation (9) [27]:

(7) $$R_{b}^{n}=R_{z}(\psi )R_{y}(\theta )R_{x}(\phi )$$

(8) $$R_{n}^{e}=R_{z}(\pi -\lambda )R_{y}(\pi /2-\varphi )$$

(9) $$R_{b}^{e}=R_{n}^{e}R_{b}^{n}$$

The required rotation matrix for image georeferencing is $R_{i}^{e}$, which describes the rotations from the camera coordinate system (*i*) to the ECEF one. The rotation matrix $R_{i}^{e}$ can be calculated using the rotation matrix $R_{b}^{e}$ and the bore-sight matrix $R_{i}^{b}$:

(10) $$R_{i}^{e}=R_{b}^{e}R_{i}^{b}$$

**Figure 4:**
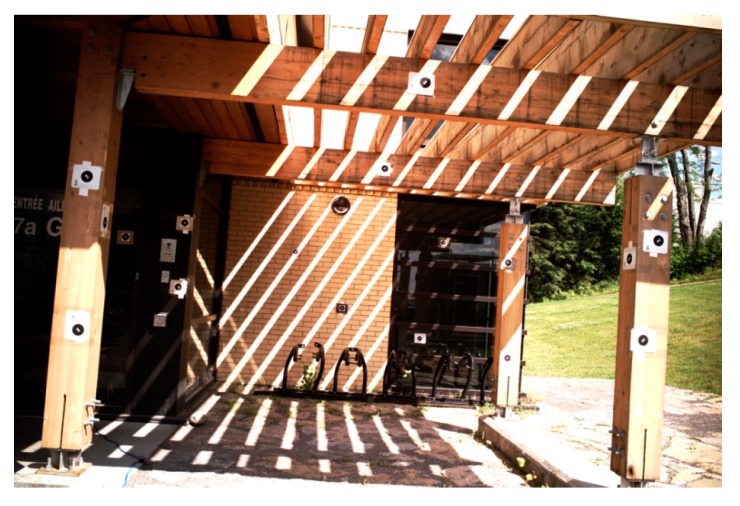
Test-field for platform calibration.

To determine the bore-sight matrix $R_{i}^{b}$, first, a network of targets is established in the ECEF coordinate system (Figure 4). Then, the targets are photographed using the camera which is firmly installed on the platform with the INS. Simultaneously, the INS data ($R_{b}^{n}$) is logged. At the post-processing stage, the position (${\vec{X}}_{i}^{e}$) and orientation ($R_{i}^{e}$) of the camera center are calculated via photogrammetric resection. Using the geodetic coordinates of the camera center $\left(\varphi_{i}^{e},\lambda_{i}^{e}\right)$—derived from Cartesian coordinates ${\vec{X}}_{i}^{e}$—the rotation matrix $R_{n}^{e}$ is calculated. Then, the rotation matrix $R_{b}^{e}$ from the INS body-fixed system to the ECEF system is determined via Equation (9). Finally, by substituting $R_{i}^{e}$ and $R_{b}^{e}$ to Equation (10), the unknown bore-sight matrix $R_{i}^{b}$ is determined.

Notice that, in this study, the camera and the INS were stacked together, in a fixed status as in Figure 1c. Consequently, the platform could be calibrated before installing the sensors on the UAV.

### 3.3. System Integration

A UAV-PS consists of a platform, camera, navigation system and control system. The control system is responsible for various tasks including power control, setting the data-acquisition parameters, data logging, data storage and time synchronization. In this study, the hardware of the control system simply includes the computer and the power supply as described in Section 2.4. The software solution developed for this control system contains three main classes: INS, camera, and clock.

The main functionality of the clock class is to get the time up to nanoseconds from the system-wide clock and assign it to any acquisition event in a real-time manner. The INS class is responsible for communication with the INS and recording the navigation messages. Each navigation message contains the information of position, rotation and GPS time. The system time at the moment of receiving each navigation message is also assigned to that message by the clock class. The GPS time of the messages is assigned to a shared variable as well, which has external linkage to the camera class. The camera class is responsible for communication with the camera and setting its attributes including triggering time interval, exposure and gain value, and frame size. Although several methods of acquisition are available for Prosilica cameras, software triggering mode is used to facilitate the synchronization process. That is the camera is triggered automatically based on defined intervals, e.g., every 500 ms. The end of camera exposure is set as an event, and a callback function is registered to this event. The functionality of this callback is to save the acquired frame and tag the navigation information to it. This information includes the GPS time and the navigation data received from the INS as well as the system time observed at the epoch of the exposure-end event. Finally, the software makes these classes operate together. It starts two threads in the calling process to execute the main functions of the classes simultaneously.

Notice that the GPS time, tagged to each image, is determined by the INS class and the frequency of INS data is 50 Hz. Therefore, the GPS timestamp is theoretically less than 20 ms different from the exact time of the exposure. Assuming a flight speed of 20 km/h, the time-synchronization error of 20 ms can cause 11 cm of shift between the true position and the tagged position of an image. When a navigation-grade GPS is used, such a shift is quite below the precision of GPS measurements and can be ignored [7]. However, if differential GPS measurements are used, then this error must be systematically handled [11]. To do so, in a post-processing step, the difference between the system time and the GPS time that are tagged to each image is used to derive the exact navigation data corresponding to that image via a linear interpolation over the two INS messages, between which the image is acquired.

## 4. Data Acquisition

**Figure 5:**
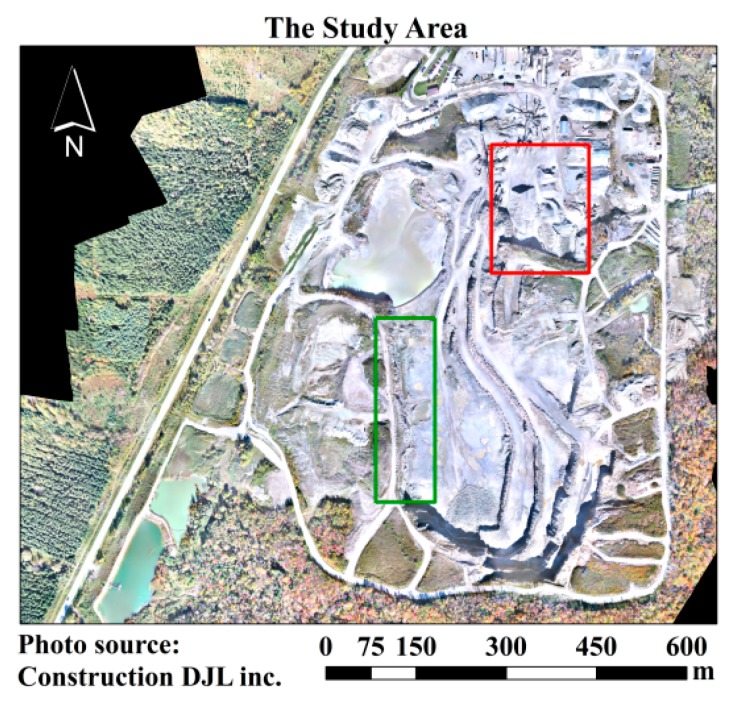
The study area and mapping zones.

In this study, the data were acquired from a gravel pit at Sherbrooke, QC, Canada. The extent of the gravel-pit mine is shown in Figure 5. Two series of data over a period of two months were acquired—August and October 2014. Two main zones were considered for the experiments (Figure 5). The red zone represents one part of the gravel pit which was covered by stockpiles, and the green zone represents the zone covered by cliffs and rocks.

### 4.1. Data-Acquisition Planning

**Figure 6:**
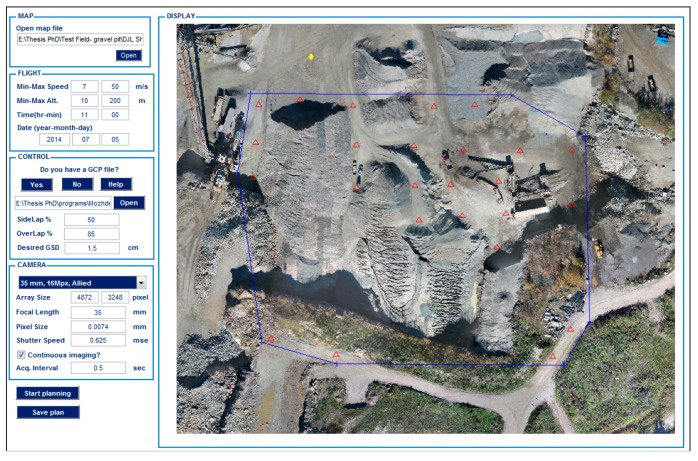
Interface of the flight-planning software.

In order to perform flight planning, there exist several software packages. However, in this study, a simple software solution is developed to satisfy the specific needs of the project for both flight and fieldwork planning. The interface of the software is shown in Figure 6. The main inputs of the software are the platform and sensor characteristics as well as the desired overlap and ground resolution for imagery. In order to calculate the position of the sun, the flight time is needed too. Knowing the position of the sun helps to minimize shadow effects; the larger the solar elevation angle, the shorter the shadows. The software allows users to either load or graphically choose the predicted positions of GCPs—red triangles in the display panel of Figure 6. It is, then, possible to determine the flight zone—blue polygon and the flight home—yellow lozenge. The software designs and logs the flight plan afterwards. One of the significant applications of this software is to design the approximate spatial distribution of GCPs considering two conditions. First, GCPs should be installed at stable locations distributed over the whole imaging zone. Second, their visibility in the images should be maximized.

### 4.2. Fieldwork

**Figure 7:**
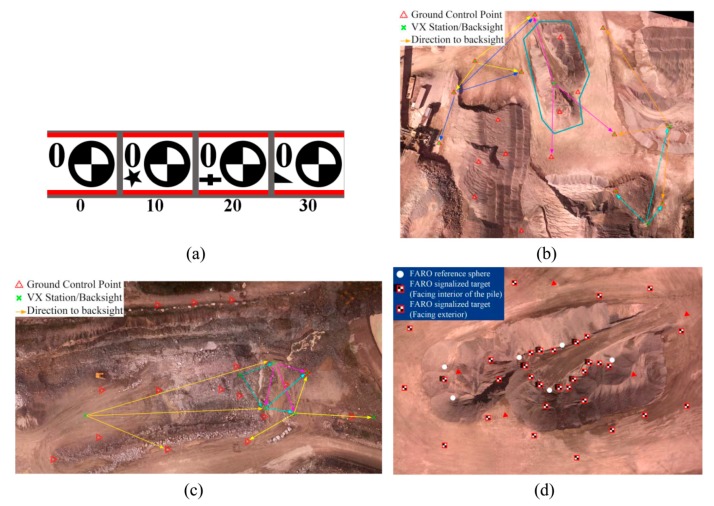
Surveying plans (**a**) Markers for GCPs; (**b**) Configuration of GCPs and laser-scanner stations for dataset A; (**c**) Configuration of GCPs and laser-scanner stations for dataset C; (**d**) Configuration of targets for FARO scanner over one pile.

The first task of the fieldwork was to initialize the GPS base receiver for collecting RTK measurements. The absolute coordinates of the base point were determined with 2–5 mm accuracy. The next step was to install the targets at locations predicted during the flight planning stage (see Section 4.1). Then, their positions were measured using the R8 GNSS System (Trimble, Sunnyvale, CA, USA)—a high-precision, dual-frequency RTK system. Following the same concept as camera calibration (Section 3.1.2), GCPs were marked as circular targets (Figure 7a). Once the GCPs were established, the flights for image acquisition started. Table 1 presents the flight conditions. Labels are given to the acquired datasets for further use in this paper.

**Table 1:** Information of the data-acquisition sessions.

Characteristic	Flight Date		
	August 2014	August 2014	October 2014
	Dataset A	Dataset B	Dataset C
**Weather temperature (°C)**	22	26	10
**Wind speed (Km/h)**	8	19	8
**Zone structure**	Stockpiles	Cliffs	Cliffs
**Approximate flight altitude (m)**	80	90	90

Upon termination of image acquisitions, the terrestrial surveying for gathering check data started. The check data included sparse 3D point clouds measured by Trimble VX laser scanner over the whole mapping area and a dense point cloud measured by a FARO Focus laser scanner (FARO, Lake Mary, FL, USA) over a small pile. Figure 7b,c show the configuration of the control points, the stations where VX scanner was installed as well as the zone where the dense 3D point cloud was measured by FARO scanner (blue polygon). In order to facilitate the accurate registration of individual FARO scans, several targets with checkerboard pattern and reference spheres were installed at different levels of the pile (Figure 7d).

## 5. Data Processing Workflow

**Figure 8:**
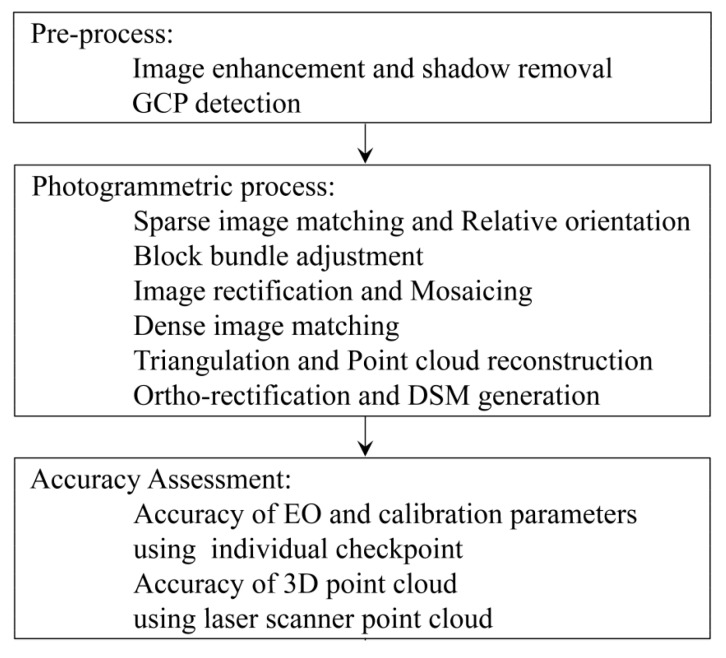
Photogrammetric workflow to produce topographic data from images.

The main steps of data processing in aerial photogrammetry are illustrated in Figure 8. The ordinary methods to perform these steps are not discussed here. Instead, the methodology of this study to improve some of these procedures is presented.

### 5.1. Image Pre-Processing

#### 5.1.1. Intensity Enhancement

**Figure 9:**
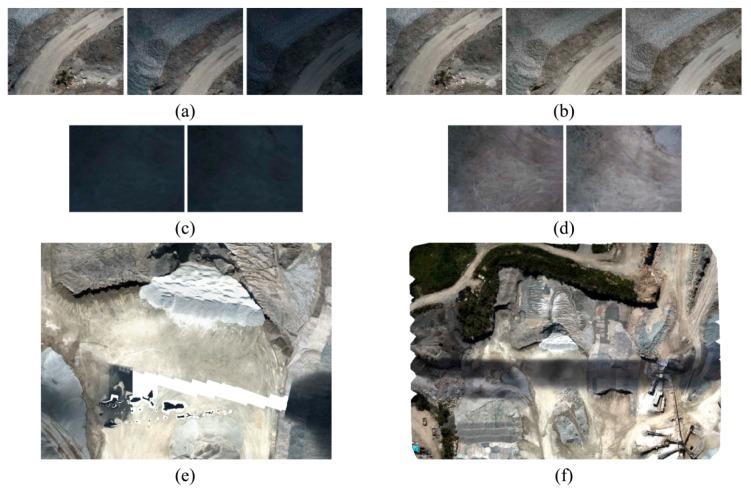
Intensity enhancement (**a**) A sequence of illuminated and dark images; (**b**) Images of Figure 9a after correction; (**c**) Two adjacent dark images with low texture diversity; (**d**) Images of Figure 9c after correction; (**e**) Failure in ortho-mosaic generation; (**f**) Correct mosaic after intensity enhancement.

As in the system of this study, in most of UAV-PSs, small-format cameras are used because of weight limitations. One of the main problems caused by such cameras is their small ground coverage. This characteristic makes the sequence of images vulnerable to photometric variations, even though the flight time is usually quite short [28]. Noticeable radiometric changes among adjacent images make both sparse matching and pixel-based dense-matching more difficult [29,30]. An example of such situation is given in Figure 9. These images are from a test dataset not listed in Table 1. They were taken with a Prosilica GT1920C camera with sensor size of 8.7894 mm × 6.6102 mm and focal length of 16 mm. As shown in Figure 9a, some images are considerably darker than their neighboring images since a patch of dark clouds had passed through the zone. Some of the images additionally suffer from lack of texture diversity (Figure 9c). As a result, matching such images becomes difficult. Therefore, their relative orientation parameters cannot be determined, and the ortho-mosaic cannot be generated either (Figure 9e).

When thematic applications are required, sophisticated techniques using spectral observations should be used for radiometric correction of images. Otherwise, simple image enhancement methods can be used to reduce the relative photometric variations. In this study, a combination of white balancing and histogram matching is proposed. Starting by the image with proper illumination as the reference image, its dark neighboring image is first white-balanced. Then, the intensity histograms of both images are calculated. If the correlation of the cumulative distribution functions of the histograms is more than 0.5, then no further enhancement is needed. Note that a cumulative histogram represents the intensity rank of each pixel with respect to other pixels regardless of general radiometric changes [31]. If the correlation of two distributions is less than 0.5, then histogram matching is performed to adjust the intensity values in the dark image. With this method, each image is relatively corrected only with respect to the images immediately adjacent to it. Therefore, the trace of dark images is still visible globally. However, this intensity enhancement makes the matching performable, and a correct ortho-mosaic can be generated (Figure 9f).

#### 5.1.2. Shadow Removal

**Figure 10:**
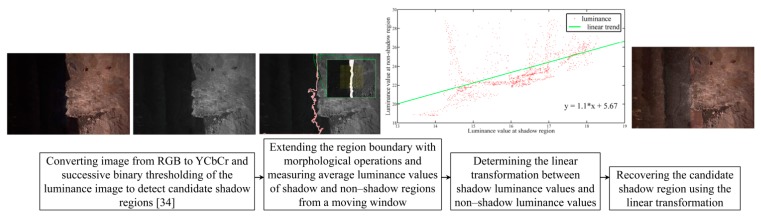
Workflow for automatic shadow detection and removal.

Another radiometric effect on aerial images is caused by shadows. In thematic applications such as atmospheric correction and classification, shadow regions lead to inevitable errors [32]. Shadows can also cause spatial errors in 3D modeling. This usually happens when the sun direction changes slightly during the flight and the shadow edges move as a consequence [33]. In our experiments, this problem was observed in dataset B (Table 1). In order to investigate the effects of shadows on the quality of 3D modeling, a simple technique is proposed to detect and remove the shadow regions from single images. The summary of this technique is presented in Figure 10. In Section 7.3, the effects of shadow removal on both the photometric appearance and the accuracy of the 3D point clouds are analyzed.

#### 5.1.3. GCP Detection

**Figure 11:**
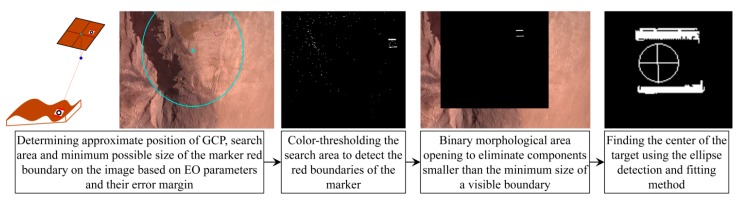
Workflow for automatic detection of GCPs.

Once the image intensities are enhanced, ground control points can be detected. Automatic detection of GCPs in images is important from two aspects. Firstly, detecting GCPs manually in large sets of UAV images is a cumbersome task. Secondly, the accuracy of target detection directly affects the accuracy of georeferencing and calibration. Therefore, more attention has recently been paid to this process [10,35]. The method applied to position GCPs is presented in Figure 11. It is mainly based on localization of GCPs using direct EO parameters, color-thresholding and ellipse detection as described in Section 3.1.2. Although this process is automatic, the results should manually be verified to remove incorrect detections.

### 5.2. Photogrammetric Processing

Photogrammetric processes are applied to aerial images in order to generate various types of topographic products such as 3D point cloud, geo-referenced mosaic and digital surface model (DSM). In this study, the main photogrammetric processes are performed using Pix4D software [36]. This software has recently been popular among researchers and commercial users for UAV-photogrammetry [33]. Several experiments were designed using this software in order to evaluate the performance of the developed UAV-PS (Section 6.2).

Furthermore, a BBA strategy is suggested for on-the-job self-calibration. This strategy is able to control the adverse effects of noisy aerial observations as well as correlation of IO and EO parameters on the accuracy of self-calibration. The main solution of this strategy is to transform the intrinsic camera calibration parameters from unknowns to pseudo-observations. That is, they should be considered as semi-unknowns with known weights. The experimental way to determine these weights is presented in Section 6.1, and the results are discussed in Section 7.6. In the following paragraphs, the principals of self-calibration with pseudo-observations are presented. Since BBA and least-squares adjustment are well-studied topics, the details are avoided here. Readers are referred to [16,37,38] for more theoretical information.

The mathematical model of bundle adjustment with additional parameters can be presented as in Equation (11), where the observation equations (*F*) are based on co-linearity condition. These equations are functions of measurements (*L*), unknowns (*Y*) and pseudo-observations (*X*). The measurements are image coordinates of tie points. The unknowns include ground coordinates of tie points and EO parameters of images. The pseudo-observations include intrinsic calibration parameters—both IO parameters and distortion terms:

(11) F(X,Y,L)=0

The linear form of Equation (11) is obtained using a Taylor series first-order approximation:

(12) W+AδK+BδL=0

where *K* is a concatenated vector by *X* and *Y*, *W* is the miss-closure matrix, and *A* and B are the matrices of first-order partial derivatives of *F* with respect to *K* and *L*, respectively*.* Assuming that $P_{L}$ is the weight matrix of the measurements, $P_{X}$ is the weight matrix of the pseudo-observations, and *D* is the matrix of datum constraints, then the least-squares solution for δK can be obtained as in the following equation:

$$\Sigma_{K}={\left(P_{X}+A^{T}{\left(B{P_{L}}^{-1}B^{T}\right)}^{-1}A+D^{T}D\right)}^{-1}-D^{T}{\left(DD^{T}DD^{T}\right)}^{-1}D$$

(13) $$\delta \hat{K}=-\Sigma_{K}(A^{T}{\left(B{P_{L}}^{-1}B^{T}\right)}^{-1}W)$$

The vector of residuals, δL , is also estimated as follows:

(14) $$\text{δ}\hat{L}=-{P_{L}}^{-1}B^{T}{\left(B{P_{L}}^{-1}B^{T}\right)}^{-1}(A\text{δ}\hat{K}+W)$$

These partial solutions, $\delta \hat{K}$ and $\delta \hat{L}$, are successively added to the initial estimations of the unknowns and values of the measurements and pseudo-observations until reaching convergence. The role of the weight matrix *P_X_* in Equation (13) is to control the changing range of pseudo-observations.

## 6. Experiments

In this section, the experiments which were performed to assess different aspects of the developed system are presented. The results obtained from these experiments are, then, discussed in Section 7.

### 6.1. Laboratory Experiments

#### 6.1.1. Calibration

The camera was calibrated several times during a period of few months before starting the data acquisition. The final parameters were obtained as the average of the parameters from these tests. The stability of each calibration parameter was also determined as its variance at these tests. In the BBA strategy of Section 5.2, these variances were used as the weights of pseudo-observations.

In order to verify the accuracy of offline calibration parameters obtained from these test, 10 check images were captured. In these images, the targets were detected using the method of Section 3.1.2. Some of them were reserved as checkpoints, and others were served as control points. Using the control points and the calibration parameters, the EO parameters of the images were determined via space resection. Then, the 3D object-space coordinates of the checkpoints were back-projected to the images. The difference between the back-projected position of a checkpoint and its actual position on an image is called the residual. The residuals show how accurate the calibration parameters are modeled. The results obtained from this test are presented in Section 7.1.

In order to analyze the efficiency of automatic target detection, compared with manual target detection, similar calibration and assessment tests were performed using the targets that were detected manually. Positions of the manual targets were different from those of the automatic targets with an average of 1.3 pixels and maximum of 2.4 pixels. The results obtained from this experiment are also presented in Section 7.1.

#### 6.1.2. Time Synchronization

In order to verify how precisely the camera exposures could be tagged via INS messages, a simple experiment was performed. For each image, the INS log file was searched, and the INS message whose GPS time was exactly equal to the GPS timestamp of the image was detected. If the system time tagged to that INS message were adequately close to the system time tagged to the image, then it could be concluded that the GPS timestamp tagged to the image was accurate too. The results of this test, performed on more than 2500 images, are discussed in Section 7.2.

### 6.2. Photogrammetric Tests

**Figure 12:**
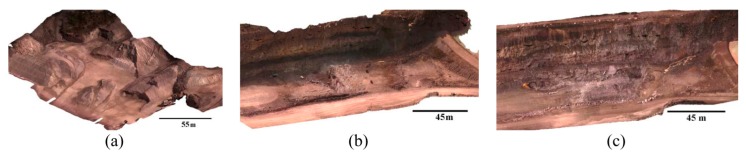
Triangulated mesh for (**a**) Dataset A; (**b**) Dataset B after shadow removal; (**c**) Dataset C.

Initially, the images were processed using Pix4D. The 3D triangulated mesh objects for dataset A, dataset B after shadow removal and dataset C are presented in Figure 12. To evaluate the accuracy of dense reconstruction, cloud-cloud comparison was performed between the image point clouds and terrestrial laser-scanner point clouds. To this end, the CloudCompare open source software was applied [39]. For each point in the laser cloud, the closest point in the image cloud was found, and the distance between them was calculated. Then, the distances were analyzed to measure the spatial accuracy of image point clouds. The results of these analyses are presented in Section 7.3. Once the accuracy of individual point clouds was assessed, they were used to produce other topographic data such as slope maps. Using the DSMs of two different dates, volumetric changes within the site were measured as well. The results are discussed in Section 7.7.

**Figure 13:**
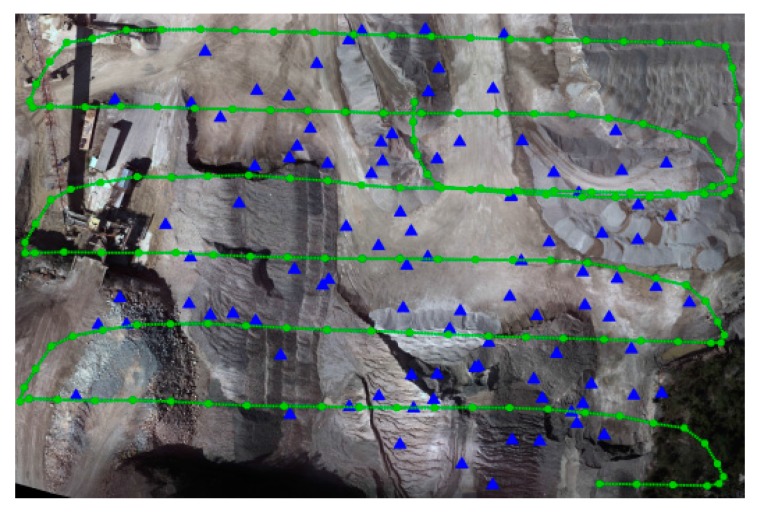
Flight trajectory and distribution of checkpoints in dataset A.

The second series of the experiments were performed to determine how the number and spatial distribution of GCPs affect the accuracy of 3D modeling. Traditionally, it is known that having more than enough GCPs with good geometrical configuration improves the accuracy of the results [37]. However, in most of UAV-mapping applications, only a minimum number of GCPs can be established. Therefore, it is important to have an *a priori* knowledge of how the final accuracy of 3D modeling would be affected by the GCPs. To this end, several experimental tests were designed. These tests are described through Table 2. In each test, the initial photogrammetric processing was performed using Pix4D, which included tie point generation, block bundle adjustment and self-calibration. Then, the accuracy of the results was evaluated against checkpoints. The checkpoints in these experiments (Figure 13) were either the GCPs not used as control points and/or some of the individual laser-scanner points that were transformed to checkpoints. To do this, the ground coordinates of each laser point were back-projected to the images via the accurate indirect EO parameters. Then, SURF feature descriptors were calculated over the projected pixels in all the images [40]. If such pixels represented salient features, and if the distances between their descriptors were smaller than a threshold, then that laser point was considered as a checkpoint. In addition, all the checkpoints were manually verified to avoid errors.

**Table 2:** Description of experimental tests for verifying the effect of number/distribution of GCPs.

Test Label	Figure	Descriptions	
GCPTest 1	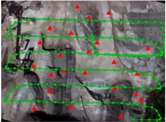	Number of GCPs	22
		Distribution	Covering the whole imaging zone
GCPTest 2	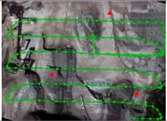	Number of GCPs	3
		Visibility *	9, 12 and 21 images
		Distribution	Evenly distributed over the imaging zone
GCPTest 3	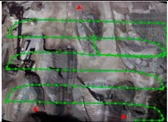	Number of GCPs	3
		Visibility	4–6 images
		Distribution	Well distributed over the imaging zone
GCPTest 4	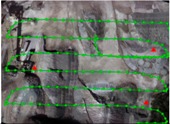	Number of GCPs	3
		Visibility	19, 20 and 22 images
		Distribution	Positioned near the ends of flight strips
GCPTest 5	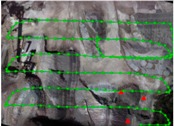	Number of GCPs	3
		Visibility	5, 12 and 21 images
		Distribution	Established at the flight home due to inaccessibility to the rest of the imaging zone
GCPTest 6	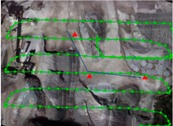	Number of GCPs	3
		Visibility	15, 17 and 22 images
		Distribution	Established along a hypothetical road due to inaccessibility to other areas

* Number of images, in which every GCP is visible.

Moreover, we were interested in assessing the results of these tests not only with GCPs that were measured by RTK GPS, but also with GCPs whose positions were measured by other techniques. Since the GCPs were originally measured only by accurate RTK GPS, it was decided to simulate the measurements for other techniques. To this end, three reference points were established outdoor, and their exact coordinates with RTK GPS system were measured. Then, other GPS devices were used to re-measure their coordinates. Using the observations made by each device, the positioning errors of GCPs were simulated. First, a Garmin GLO-GPS was used, and more than 10,000 observations were recorded over the reference points. This device is WAAS-enabled and receives position information from both GPS and GLONASS satellites. The root mean square (RMS) positioning error for this device was 2.40 m horizontally and 6.04 m vertically. Similarly, a series of 2000 observations were made with a SXBlueII GPS. This device is also WAAS-enabled and performs additional code-phase measurements and multi-path error reduction. The RMS positioning error with this device was 0.65 m horizontally and 0.69 m vertically. The results obtained in different tests using these types of GCPs are presented in Section 7.4.

**Figure 14:**
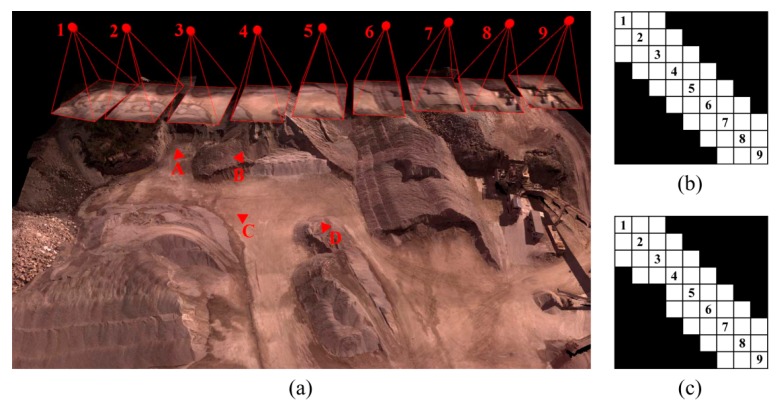
(**a**) Experiment to assess the effect of imaging configuration; (**b**) Connectivity matrix in OverlapTest 1; (**c**) Connectivity matrix in OvelapTest 2.

To analyze the effect of imaging configuration in absence/presence of GCPs on the accuracy of 3D modeling, a sequence of nine images was considered (Figure 14a). Ground control points A, B and C were visible in images 1–4, while point D was visible in images 5–6. The following situations were, then, designed and tested.

- OverlapTest 1: Each image was overlapped with at least three connected images. For example, image 5 had common tie points with both images 3 and 4. Such a connection is illustrated via the connectivity matrix in Figure 14b.
- OvelapTest 2: The situation was the same as OverlapTest 1. However, image 5 did not have any common tie points with both images 3 and 4; *i.e.*, it was only overlapped with image 4. The connectivity matrix of Figure 14c shows this situation.

The objective of these tests was to find out whether the lack of overlap in OverlapTest 2 could cause problems in BBA, and how important the role of GCPs was to solve those problems. The results obtained from each test and the issues involved with each situation are assessed in Section 7.5.

The final series of photogrammetric experiments were performed to analyze the effect of on-the-job self-calibration on the accuracy of IO and EO parameters. Also, the proposed self-calibration strategy of Section 5.2 was evaluated, and the results were compared with those of traditional self-calibration. To this end, the following situations were considered, and correlation analysis was performed at each situation in order to determine the dependency of IO parameters to EO parameters.

**Figure 15:**
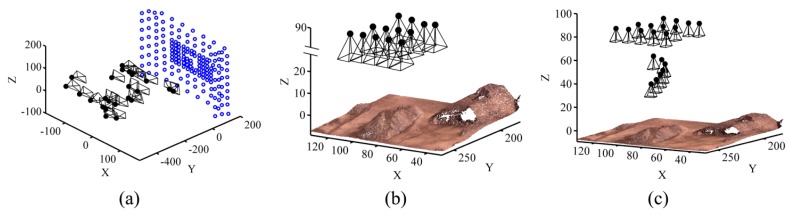
Self-calibration experiments (**a**) CalibTest 1; (**b**) CalibTest 2; (**c**) CalibTest 3.

- CalibTest 1: Offline camera calibration was performed using a well-configured imaging network (Figure 15a).
- CalibTest 2: On-the-job calibration was performed using typical aerial images, which were all acquired from almost the same altitude (Figure 15b).
- CalibTest 3: On-the-job calibration was performed using aerial images, which were acquired from varying altitudes (Figure 15c).

## 7. Results and Discussion

### 7.1. Calibration Results

**Figure 16:**
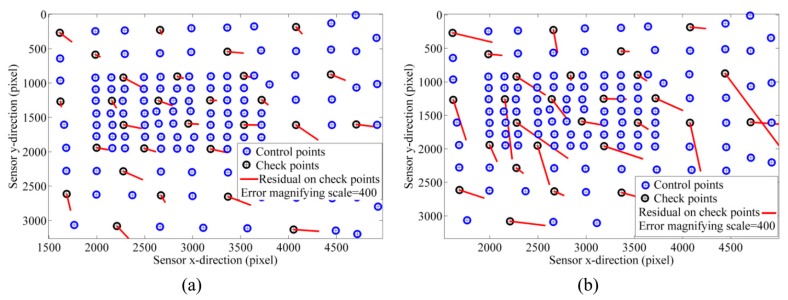
Calibration results (**a**) Residuals on checkpoints based on automatic target detection; (**b**) Residuals on checkpoints based on manual target detection.

Figure 16a shows the residual vectors for the checkpoints after camera calibration. The mean and standard deviation (StD) of the residuals on the checkpoints at *x*- and *y*-directions are 0.32 ± 0.18 pixel and 0.20 ± 0.16 pixel, respectively. Figure 16b presents the residual vectors based on the manual target detection. Notice that the targets on check images were detected automatically and, only, the targets used for calibration were measured manually. As a result, the automatic target detection improves the accuracy of calibration 81.25% in comparison with the noisy observations based on manual detection even though the noise level does not exceed 2.4 pixels.

### 7.2. Precision of Time-Synchronization

**Figure 17:**
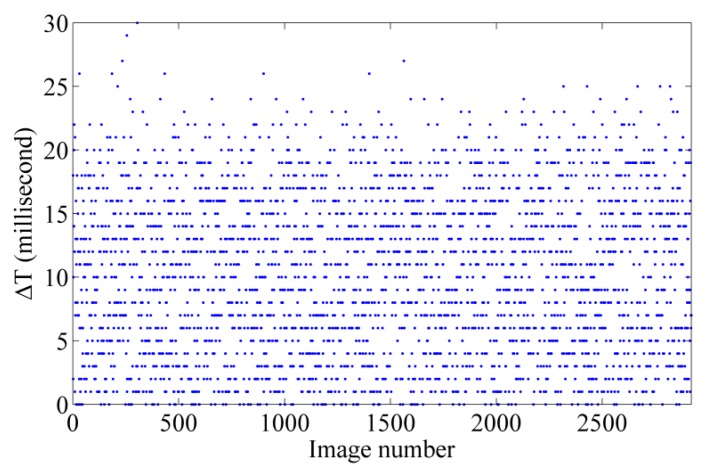
Results of the time-synchronization test.

Figure 17 demonstrates an example of the results obtained from the time-synchronization test. The *x*-axis shows the image number. The *y*-axis shows ΔT, which is the absolute difference between the system time tagged to each image and the system time tagged to its corresponding INS message. It is, indeed, the difference between the real exposure-end time of an image and the GPS timestamp tagged to it. As it can be noticed, these differences are random; however, they do rarely exceed 20 ms. This is due to the fact the INS frequency is 50 Hz (1 message per 20 ms). The main reason why this difference (ΔT) is random is that the camera exposures do not start on very exactly fixed intervals—e.g., every 500 ms. Instead, there is a few milliseconds of random delay/advance from the defined interval—e.g., 502 ms. In average, it can be concluded that the GPS timestamp tagged to any image is approximately 11 ± 7 ms delayed/advanced from the exact time of the exposure.

### 7.3. Accuracy of 3D Point Clouds

**Figure 18:**
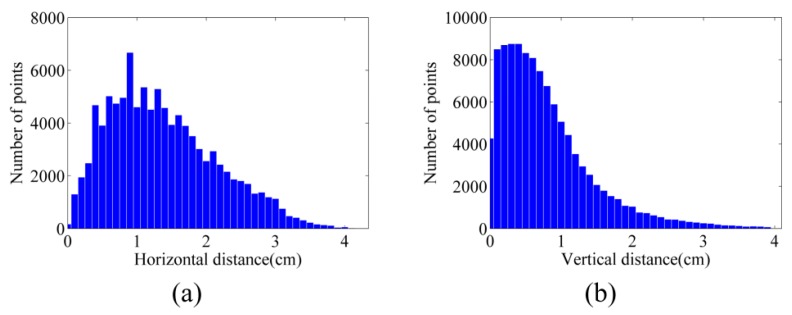
Histograms of distances between the point clouds from dataset A and laser scanner (**a**) Horizontal distances; (**b**) Vertical distances.

As mentioned in Section 6.2, image point clouds were compared with terrestrial laser-scanner point clouds. Figure 18a,b illustrate the histograms of horizontal and vertical distances between the point cloud of dataset A and that of the laser scanner (see Table 3 as well). The vertical accuracy of this point cloud is 1.03 cm, and its horizontal accuracy is 1.58 cm.

**Table 3:** Summary of distances between the image-based point clouds and laser-scanner ones.

Dataset	Horizontal Distance (cm)			Vertical Distance (cm)		
	Mean	RMS	StD	Mean	RMS	StD
**A**	1.38	1.58	0.77	0.80	1.03	0.66
**B before shadow removal**	1.79	2.03	0.96	1.41	1.72	0.99
**B after shadow removal**	1.62	1.82	0.83	1.32	1.62	0.95
**C**	1.88	2.07	0.84	1.63	2.02	1.18

**Figure 19:**
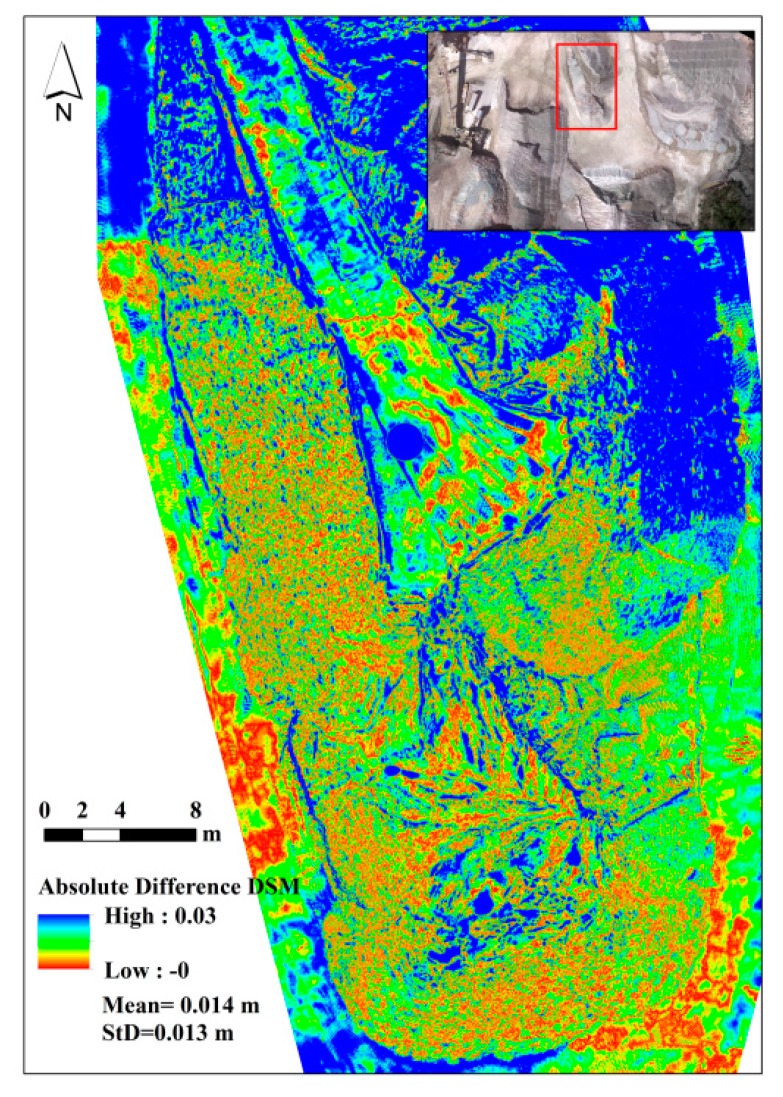
Absolute difference between the DSM from FARO laser scanner and that of dataset A.

As another test, the dense point cloud measured by FARO laser scanner was transformed to a raster DSM. Then, the absolute difference of the image-based DSM from the laser DSM was calculated. Figure 19 presents the vertical difference between the image-based DSM and laser DSM. In more than 78% of the zone, this vertical difference is less than 1 cm.

**Figure 20:**
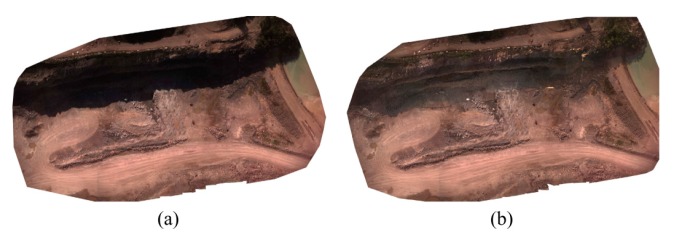
Image mosaics of dataset B (**a**) Before shadow removal; (**b**) After shadow removal.

For dataset B, as mentioned in Section 5.1.2, shadow regions were removed from the images. Figure 20a,b show the geo-referenced mosaics of the site before and after shadow removal, respectively. As it can be seen, the results are visually improved. The shadow-free mosaic can be used in thematic applications where shadow effects cause errors.

**Figure 21:**
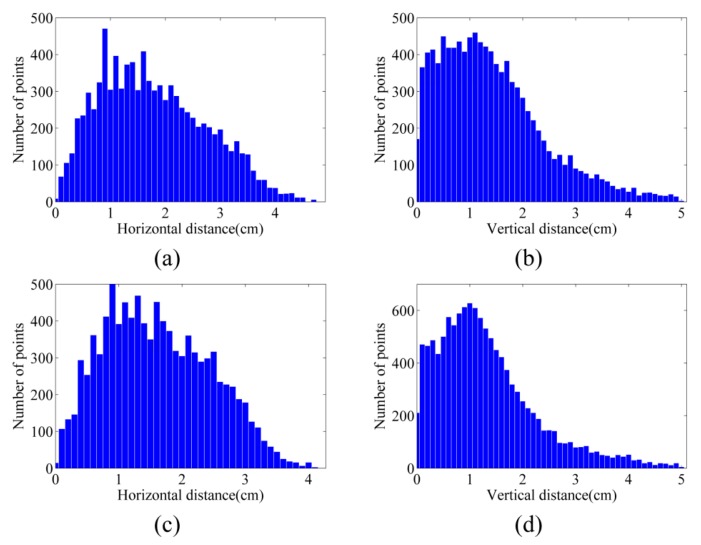
Histograms of distances between the point clouds from dataset B and laser scanner (**a**) Horizontal distances before shadow removal; (**b**) Vertical distances before shadow removal; (**c**) Horizontal distances after shadow removal; (**d**) Vertical distances after shadow removal.

**Figure 22:**
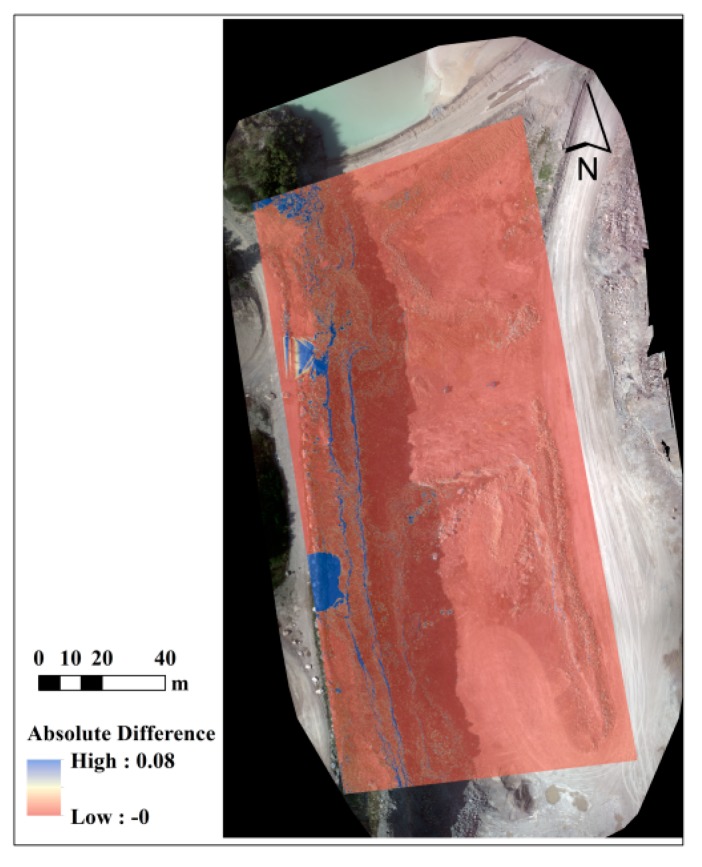
Absolute difference between the DSMs of dataset B before and after shadow removal.

The point clouds obtained before and after shadow removal were also evaluated using the laser-scanner data. The results are presented in Table 3 and Figure 21. According to the results, the vertical accuracy is improved from 1.72 cm to 1.62 cm with shadow removal. Therefore, no noticeable improvement can be observed via this test. This is principally due to the fact that the terrestrial laser-scanner points over the shadow region were not dense enough. However, when the DSMs before and after shadow removal were compared, the differences could be observed more clearly. As shown in Figure 22, large vertical differences, as large as 8 cm, can be observed in edges of the cliffs. These are the zones where more than one shadow was casted on the objects—the shadow from two higher rows of the rocks. It is believed that this type of shadow causes errors in the dense matching and 3D reconstruction process.

**Figure 23:**
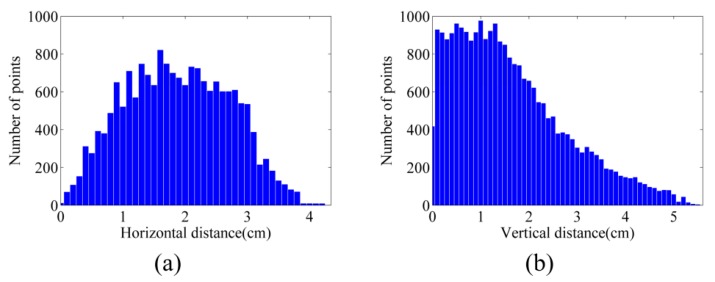
Histograms of distances between the point clouds from dataset C and laser scanner (**a**) Horizontal distances; (**b**) Vertical distances.

Finally, the point cloud of dataset C was evaluated against the terrestrial laser point cloud. The results are presented in Table 3 and Figure 23. Generally, the accuracy of the point cloud from dataset A is higher than both dataset B and dataset C. This could be explained by the structure of the stockpiles in dataset A, where less occlusion happens in aerial images. For datasets B and C, the vertical and layered structure of the cliffs causes more errors.

### 7.4. Effects of Ground Control Points

Firstly, the results obtained via direct georeferencing are discussed. In fact, DG may be interpreted in two senses. In the first sense, direct EO parameters of images from the navigation data are directly used for 3D modeling without any photogrammetric refinement applied to them. In this case, offline intrinsic camera calibration parameters should be used as no on-the-job self-calibration is performed. This strategy is mostly used for rapid mosaicking and ortho-photo generation. The results from this test for dataset A are shown as DG type 1 in Table 4. In the second sense, direct navigation data are used as inputs into initial photogrammetric processing and the EO parameters are slightly refined within a free-network adjustment. Then, these refined EO parameters are used for 3D modeling. The results from this test are shown as DG type 2 in Table 4. Notice that in this table and the following ones, the mean error represents the average of absolute errors—not the signed ones. As it can be seen, the horizontal and vertical accuracy is improved 31% and 73%, respectively, after applying the initial processing. The main reason for this improvement is that all the relative-orientation errors between images are corrected within the initial processing. This can be observed from the fact that standard deviations of errors decrease considerably after initial processing.

**Table 4:** Accuracy of direct georeferencing on checkpoints.

Experiment	X-Direction Error (m)			Y-Direction Error (m)			Vertical Error (m)		
	Mean	RMS	StD	Mean	RMS	StD	Mean	RMS	StD
**DG type 1**	1.146	1.406	0.837	2.478	3.088	1.892	10.440	11.670	5.359
**DG type 2**	1.938	1.943	0.137	1.162	1.166	0.090	3.159	3.169	0.268

The results obtained from the experimental tests with GCPs (Table 2, Section 6.2) are presented in Table 5. For each checkpoint, the EO and calibration parameters of images—after indirect geo-referencing at each experimental test—were used to determine its ground coordinates via intersection. Then, the error on the checkpoint was measured as the difference between its ground-truth 3D coordinates and the calculated coordinates.

In order to provide a better understanding of the way each configuration or device affects the results, the relative changes of accuracy are represented in Table 6. These change rates are calculated as the percentage of RMS improvement with regard to the lowest accuracy. Therefore, the improvement rate of 0.0% shows the reference value used for change-percentage measurement.

**Table 5:** Horizontal accuracy on checkpoints based on different GCP experiments.

Error	Experiment	Trimble R8			SXBlue			Garmin GLO		
		Mean	RMS	StD	Mean	RMS	StD	Mean	RMS	StD
**Horizontal Error (cm)**	**GCPTest 1**	0.2	0.4	0.3	61.9	61.9	3.0	180.0	180.7	12.4
	**GCPTest 2**	0.3	0.4	0.6	68.0	69.0	1.7	158.2	160.8	19.6
	**GCPTest 3**	0.8	0.9	1.2	73.9	74.1	4.6	216.4	216.6	9.0
	**GCPTest 4**	0.3	0.4	0.7	63.8	62.9	2.3	160.2	165.3	30.2
	**GCPTest 5**	0.6	0.8	0.9	74.7	76.3	14.8	227.0	228.0	20.5
	**GCPTest 6**	0.3	0.5	0.6	72.6	72.8	5.1	189.3	193.5	31.4
**Vertical Error (cm)**	**GCPTest 1**	1.2	1.7	1.2	13.8	15.5	7.0	412.9	413.0	10.5
	**GCPTest 2**	1.6	2.0	1.2	41.1	49.7	28.3	355.5	355.8	12.4
	**GCPTest 3**	4.1	4.3	1.4	73.6	73.6	2.8	434.9	436.0	32.5
	**GCPTest 4**	1.4	2.0	1.4	43.2	48.5	22.1	432.1	433.5	35.2
	**GCPTest 5**	2.4	3.0	1.8	121.6	147.1	83.6	431.2	446.1	115.6
	**GCPTest 6**	1.4	1.9	1.4	80.2	97.4	55.7	432.1	433.5	35.2

**Table 6:** Improvement rate of accuracy on checkpoints based on different GCP experiments.

Device	Horizontal-Accuracy Percentage Change						Vertical-Accuracy Percentage Change					
	Experiment						Experiment					
	1 *	2	3	4	5	6	1	2	3	4	5	6
**R8 RTK**	99.8	99.8	99.6	99.8	99.6	99.8	99.6	99.6	99.0	99.6	99.3	99.6
**SXBlue**	72.9	69.7	67.5	72.4	66.5	68.1	96.5	88.9	83.5	89.1	67.0	78.2
**Garmin GLO**	20.7	29.5	5.0	27.5	0.0	15.1	7.4	20.2	2.3	2.8	0.0	2.8

* Reads as GCPTest 1.

As the results show, in order to reach the highest accuracy, it is recommended to provide a large number of well-distributed GCPs (as in GCPTest 1). However, if this is not possible, then the best solution is to install the GCPs at different sides of the imaging zone, where they can also be visible in as many images as possible (as in GCPTest 2). To ensure this condition, the best practice is to install them near the ends of the flight strips so that they are visible in several images from two adjacent strips (as in GCPTest 4). Typically, it is preferred to install GCPs at places with height variation. However, the results from GCPTest 6, where the control points are almost at the same elevation, are much more accurate than those of GCPTest 3, where GCPs have high height variation but low visibility. Finally, the least accurate results are obtained from GCPTest 5, where the GCPs are positioned at the flight home. Therefore, this solution should be avoided unless there is no other possibility. Besides, in this situation, it should be ensured that the GCPs can be commonly visible in at least three images. In order to plan any of these situations, the flight-planning software (Section 4.1) can be used.

### 7.5. Effects of Imaging Configuration

The above-mentioned experiments prove that careful application of minimum GCPs can also yield a high level of modeling accuracy. However, such accuracy level is only achievable if images provide a stable imaging and network configuration. The importance of this fact is analyzed based on the overlap tests described in Section 6.2.

**Figure 24:**
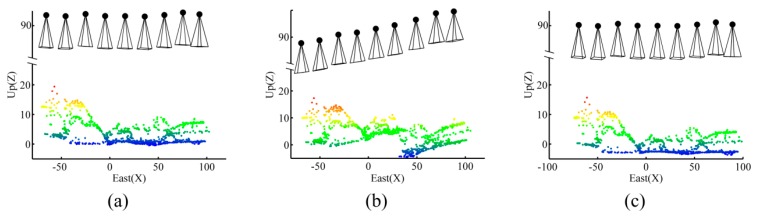
Effect of imaging configuration (**a**) Inner-constrained adjustment based on OverlapTest 1; (**b**) Inner-constrained adjustment based on OverlapTest 2; (**c**) Controlled adjustment based on OverlapTest 2 using four GCPs.

When performing BBA, the coordinate datum requires seven defined elements to compensate its rank deficiencies, namely scale, position and rotation. These defined elements can be provided with either minimum constraints in controlled networks or inner constraints in free networks. When enough overlap exists among images, both free and controlled network adjustments can be performed correctly without facing any additional rank deficiencies. Figure 24a shows the orientations of cameras and ground coordinates of tie points calculated correctly in a free network based on OverlapTest 1.

However, only one image not having enough overlap with its adjacent ones can disturb this ideal configuration. As in OverlapTest 2, image 5 does not have any common tie point with image 3. Therefore, there is no tie point to make a connection between one part of the network including images 1–4 and the other part of the network including images 5–9. Notice that this disconnection happens even though image 5 and image 4 have common tie points. As a result, the coordinate datum faces eight rank deficiencies—one additional scale deficiency. In order to resolve this, one more constraint is required. If no ground-truth measurement is available, then the solution is to assign an arbitrary scale factor to one of the unknowns. Figure 24b illustrates the results by assigning a scale factor based on the DG data to one of the tie points between images 4 and 5. In this situation, although the BBA can be solved, a wrong scale change is introduced between the two parts of the network. As a result, this solution must be avoided unless the DG data are very accurate. The practical solution to this problem is to add the ground observations of control points to the adjustment. In this example, control point D provides the additional scale constraint required to solve the 8th rank deficiency of the datum (Figure 24c). It can be concluded, that configurations of both the terrestrial data (GCPs or any other types of ground measurements) and the aerial images decide the final accuracy of 3D modeling.

### 7.6. On-the-job Self-Calibration Results

As the results in Section 7.1 show, noisy image observations affect the results of self-calibration to a great extent even if the noise level is very low. Similarly, on-the-job self-calibration of aerial images is affected by the noise in images, which is usually inevitable in UAV imagery. Another factor that affects the accuracy of on-the-job self-calibration is the particular configuration of aerial network. That is the images are acquired from a relatively fixed altitude. In fact, this network configuration reduces the numerical stability of calibration in terms of the increase in the correlation between the unknown parameters. Especially, IO parameters—the principal point offset and focal length $\left(x_{p},y_{p},f\right)$—become strongly correlated with EO parameters—the position of the camera center $\left(C_{x},C_{y},C_{z}\right)$. As a result, intrinsic calibration parameters become physically meaningless since they become dependent parameters that change relatively with the changes of EO parameters.

**Figure 25:**
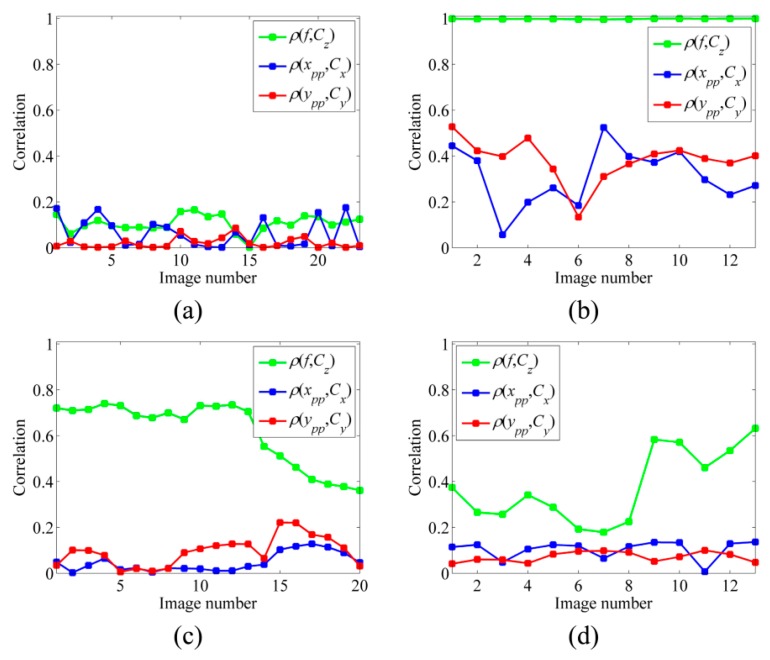
Correlation analysis in self-calibration based on (**a**) CalTest 1; (**b**) CalTest 2; (**c**) CalTest 3; (**d**) CalTest 2 by applying the proposed BBA strategy.

This effect can be practically controlled in close-range photogrammetry by providing various orientations and object depth levels as in CalTest 1. Figure 25a presents the correlation analysis of the self-calibration based on these images. As it is noticed, this condition results in very low correlation between IO and EO parameters. However, the same analysis for on-the-job self-calibration based on aerial images of CalTest 2 presents very high correlation between IO and EO parameters, specifically between focal length and imaging depth (Figure 25b).

One of the advantages of UAVs is that they can fly obliquely and at very low altitudes. Therefore, it is possible to provide more orientation variations as in CalTest 3. As a result, the average correlation between focal length and imaging depth can be reduced 38% by this new configuration (Figure 25c). However, such maneuvers are not possible in all the UAV mapping applications. Therefore, the solution proposed in Section 5.2 can be applied to improve the self-calibration. This strategy reduces the correlation between the unknowns without the need to change the network configuration (Figure 25d). For instance, the average correlation between focal length and imaging depth is reduced 60%.

### 7.7. Application-Dependant Results

**Figure 26:**
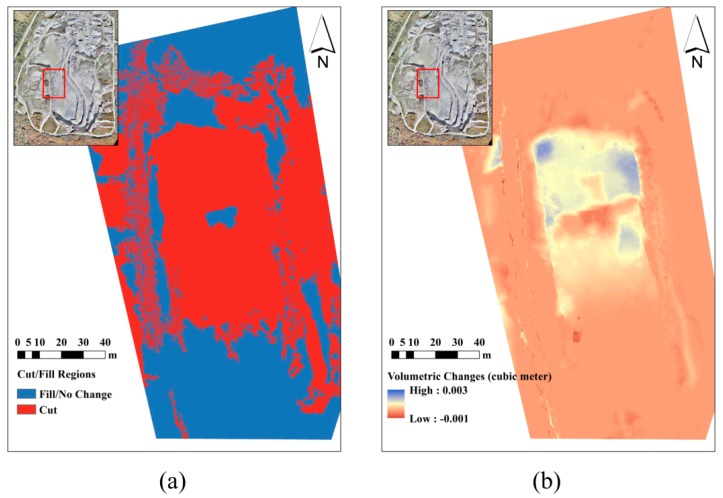
(**a**) Cut/fill regions; (**b**) Volumetric change measurement.

**Figure 27:**
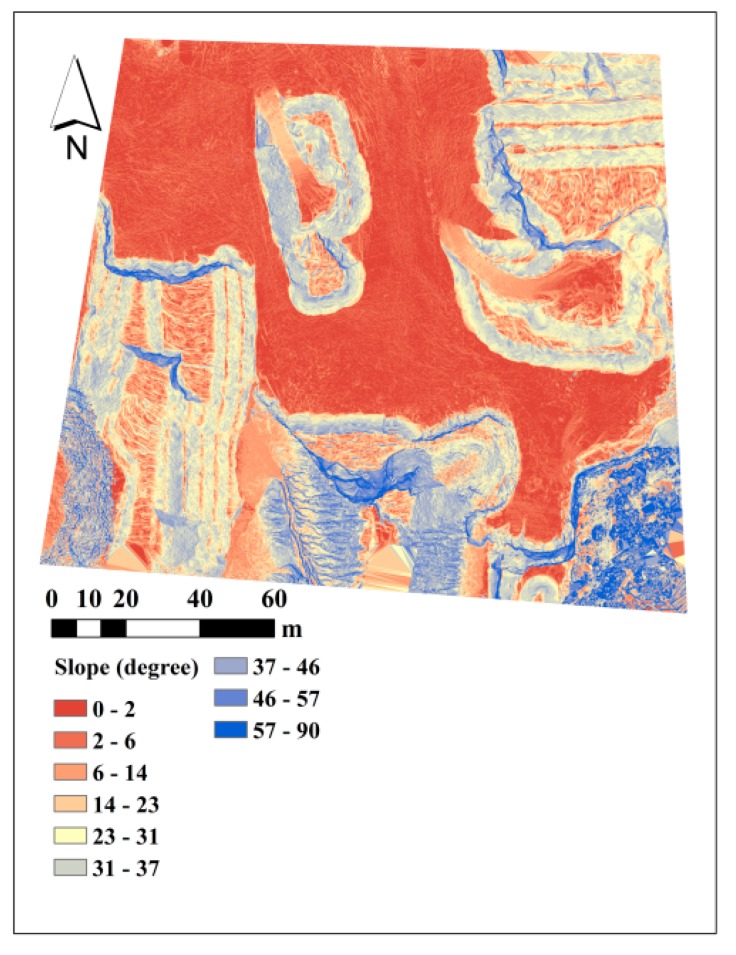
Classified slope map based on dataset A.

Figure 26a presents the major cut/fill regions based on dataset B and dataset C that were gathered with an interval of two months. As expected, most places at this zone were excavated. In Figure 26b, the volumetric change per cell is measured for every cell of the DSM. The volumetric change is measured as the difference of elevation in the before-DSM from the after-DSM which is multiplied by the cell area (1.69 cm^2^). Therefore, positive values represent excavation or cut, and negative values represent fill. The vertical accuracies of dataset B (before) and dataset C (after) are 1.32 cm and 1.63 cm, respectively. Therefore, the volumetric change measurement at each cell is performed with accuracy of 3.54 cm^3^. Figure 27 presents the slope map based on dataset A. As it can be seen, very detailed slope information is extractable from such a map, which can be used in various geological applications.

## 8. Conclusions

Various aspects of the development and implementation of a UAV-PS were discussed in this study. These included the camera offline calibration, platform calibration, system integration, flight and fieldwork planning, data acquisition, photogrammetric processing and application in open-pit mine mapping. Based on the experiments, it was concluded that the accuracy of 3D modeling with the system, either in terms of the accuracy of indirect georeferencing or the spatial accuracy of the point clouds, was better than 2 cm.

In addition to general photogrammetric experiments, several tests were performed to analyze the specific issues of UAV-based 3D modeling, and solutions were proposed to address them. It is hoped that the lessons learnt from these experiments give a more clear insight of the capacities of UAV-PSs for the upcoming studies and applications. In brief, the impact of automatic target detection on the accuracy of camera calibration was investigated. It was shown that an improvement of 81% in the accuracy of calibration could be achieved with our target detection technique in comparison with manual target detection. Regarding the system integration, it was validated that the developed software package was capable of synchronizing the navigation and imaging sensors with an approximate delay of 11 ms without requiring any additional mechanism. Moreover, the impacts of high photometric variations among images and shadowed regions on the accuracy of 3D modeling were verified. Besides, the use of a BBA strategy was suggested to improve the accuracy of on-the-job self-calibration by reducing the correlation of intrinsic camera calibration parameters to other BBA elements such as EO parameters. It was shown that, using this strategy, the correlation of IO and EO parameters could be reduced by 60% in an unsuitable imaging network. This strategy can be used in applications where the accurate, on-the-flight intrinsic calibration parameters are required independently. Furthermore, several experiments were performed to assess the effect of GCPs configuration on modeling accuracy. It was shown that a minimum number of GCPs could provide a high accuracy level if they were distributed evenly over the whole zone and their visibilities in images were maximized. However, under such conditions, the scale consistency of the imaging network needed to be ensured by providing high overlap among images.
